# Birth rate decline, COVID-19 pandemic, and vaccination programmes

**DOI:** 10.1093/hropen/hoae067

**Published:** 2024-11-12

**Authors:** Hinpetch Daungsupawong, Viroj Wiwanitkit

**Affiliations:** Private Academic Consultant, Phonhong, Lao People’s Democratic Republic; University Centre for Research & Development Department of Pharmaceutical Sciences, Chandigarh University Gharuan, Mohali, Punjab, India

Sir,

The recent *Human Reproduction Open* publication ‘Birth rate decline in the later phase of the COVID-19 pandemic: the role of policy interventions, vaccination programmes, and economic uncertainty’ by Winkler-Dworak *et al.* (2024) offers an intriguing perspective on the causes of the drop in birth rates that followed the COVID-19 pandemic in high-income nations. Nevertheless, depending solely on data from the entire population could obscure significant demographic trends that are crucial for comprehending variations in the fertility rate. For instance, different populations may have considerably different effects on fertility decisions depending on factors like age, birth order, and socioeconomic level. To obtain a more complete understanding of reproductive choices made during the crisis, these issues deserve greater research. By breaking down the data to examine the ways in which these demographic factors affected birth patterns, Winkler-Dworak et al. (2024) could have enhanced their study.

Furthermore, although Winkler-Dworak *et al.* (2024) note that vaccination programmes and economic instability are significant factors influencing birth rates, it is still unknown how exactly these factors influence birth rates. The article discusses inflation and institutional trust, but not psychological and emotional factors that could also influence family planning in unpredictable times. For instance, how does a decision to have children impact people’s views of the future and social security? Did the shifting of public health messaging during the pandemic’s waves also affect people’s attitudes on starting a family? A closer look at these underlying causes may offer further insightful background information on the trends that have been observed.

The topic of how policymakers might adapt their policies to various situations is raised by the study’s conclusion that policy interventions have varying effects on birth rates depending on institutional trust (Winkler-Dworak *et al.*, 2024). The authors could have examined how various degrees of faith in the public health system and the government can influence birth rate stabilization initiatives in times of public health emergency. What kinds of support systems and communication channels, for instance, can foster trust and have a favourable impact on reproductive decision-making? Underlining the need for contextually appropriate public health policies could be a key topic for further investigation.

Finally, even though Winkler-Dworak et al. (2024) studied birth trends from November 2020 until October 2022, it is still unknown what the long-term consequences of the post-pandemic drops in birth rates will be. Subsequent studies may concentrate on tracking birth rates for extended durations to investigate if the present patterns indicate lasting shifts or stay constant as communities adapt to the post-COVID-19 environment. Additionally, further research could investigate which social support programs—like paid time off, daycare availability, or financial incentives—work best to encourage higher birth rates in the future. Investigating these areas may suggest innovative solutions for high-income nations dealing with decreased fertility, which could lead to more resilient population landscapes.
